# Penile Pyoderma Gangrenosum: A Rare Presentation and Review of Management Strategies: Case Report

**DOI:** 10.1002/ccr3.70957

**Published:** 2025-10-08

**Authors:** Shukrani John Ngereja, Charles John Nhungo, Ezekiel Petro, Thobias Mvungi, Ibrahim Ndolla, Muhaji Mohamed, Erasto Wambura, Mukama Kasori, Fransia Arda Mushi, Hamis Isaka, Obadia Nyongole, Charles Mkony

**Affiliations:** ^1^ Department of Surgery, School of Clinical Medicine Muhimbili University of Health and Allied Sciences Dar es Salaam Tanzania; ^2^ Department of Surgery Muhimbili National Hospital‐Mloganzila Dar es Salaam Tanzania

**Keywords:** corticosteroid treatment, genital inflammatory disorders, genital ulcers, neutrophilic dermatosis, penile pyoderma gangrenosum

## Abstract

Penile pyoderma gangrenosum is a rare but important differential diagnosis for ulcerative lesions of the genital region. Early recognition and systemic treatment, especially with corticosteroids, are crucial for effective management and avoiding the penile mutilating effect of the disease. Clinicians should consider PPG in cases of unexplained genital ulcers, particularly in the absence of infection or trauma.

## 1 Introduction

Pyoderma gangrenosum (PG) is a rare, ulcerative cutaneous disorder, typically affecting the lower limbs, but it can involve other regions, including the genitalia [1]. It is a neutrophilic dermatosis characterized by painful ulcers with undermined borders [2]. Penile involvement (PPG) is extremely uncommon, with fewer than 30 cases reported. Misdiagnosis can lead to delayed treatment and penile mutilation [3]. PG can present in various subtypes, including classical, pustular, bullous, and vegetative forms. The classical subtype, which accounts for approximately 85% of all PG cases, presents with painful ulcers with a violaceous or erythematous border and an undermining edge. While PG most commonly affects the lower extremities, it can occasionally involve the penis, where it may be easily misdiagnosed and mismanaged, potentially leading to significant penile mutilation [4].

Penile pyoderma gangrenosum (PPG) is an even rarer manifestation of this condition, with fewer than 30 reported cases in the literature. PPG is characterized by painful, rapidly progressing ulcers that can mimic infection or malignancy, complicating diagnosis and treatment. This condition is frequently associated with systemic diseases such as inflammatory bowel disease (IBD), rheumatoid arthritis, and hematologic malignancies, but it can also occur in the absence of any underlying systemic illness, further complicating the diagnostic process [2].

This case report presents a rare presentation of PPG in a 45‐year‐old male patient and provides a review of the current literature on the condition, with a focus on its pathophysiology, diagnostic challenges, and management strategies.

## 2 Case Presentation

A 45‐year‐old male presented with multiple leg ulcers for 18 months, a penile ulcer for 3 months, and difficulty voiding while standing for 2 months. The leg ulcers began as painful pustules that coalesced into ulcers. The penile lesion started as a papule, eventually involving the entire shaft, mons pubis, and ventral scrotum. Over time, the glans became necrotic, and the distal two‐thirds of the penile urethra had sloughed off, leaving a stump.

There were no systemic symptoms or relevant history of STIs, diabetes, or immunosuppression.

**FIGURE 1:**
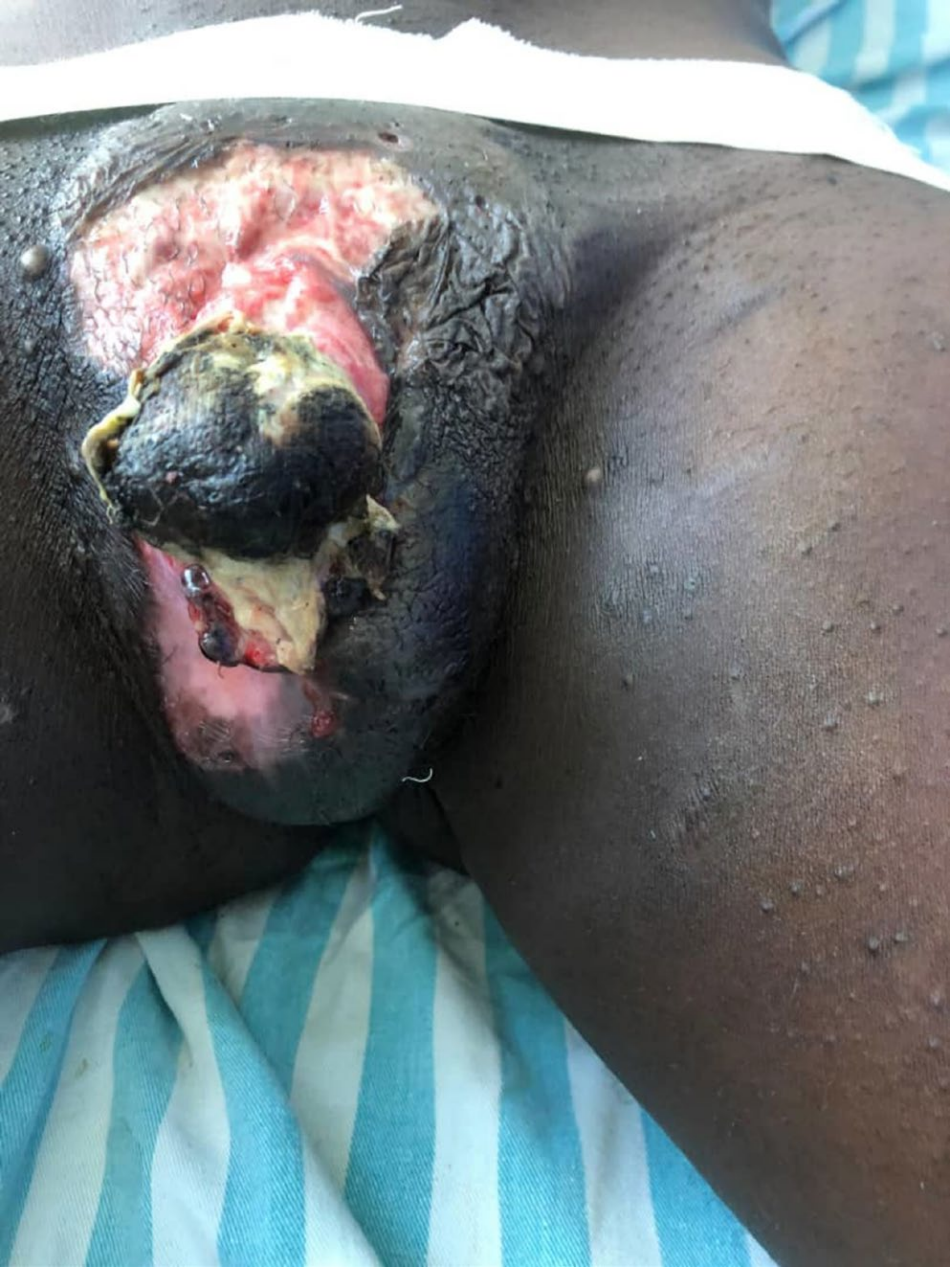
Gross photography depicting a large genital ulcer extending from the distal shaft of the penis to the mons pubis and ventral scrotal skin with gangrenous glans penis and necrotic surrounding skin.

On examination, the genital region revealed a large ulcer extending from the distal shaft of the penis to the mons pubis and ventral scrotal skin. The glans penis was gangrenous and insensate. Surrounding tissue was necrotic, and the ulcer floor was covered by pale granulation tissue, slough, and purulent discharge. The urethral defect extended proximally, leaving a visible stump near the root of the penis. No inguinal lymphadenopathy was noted (Figure 1).

**FIGURE 2:**
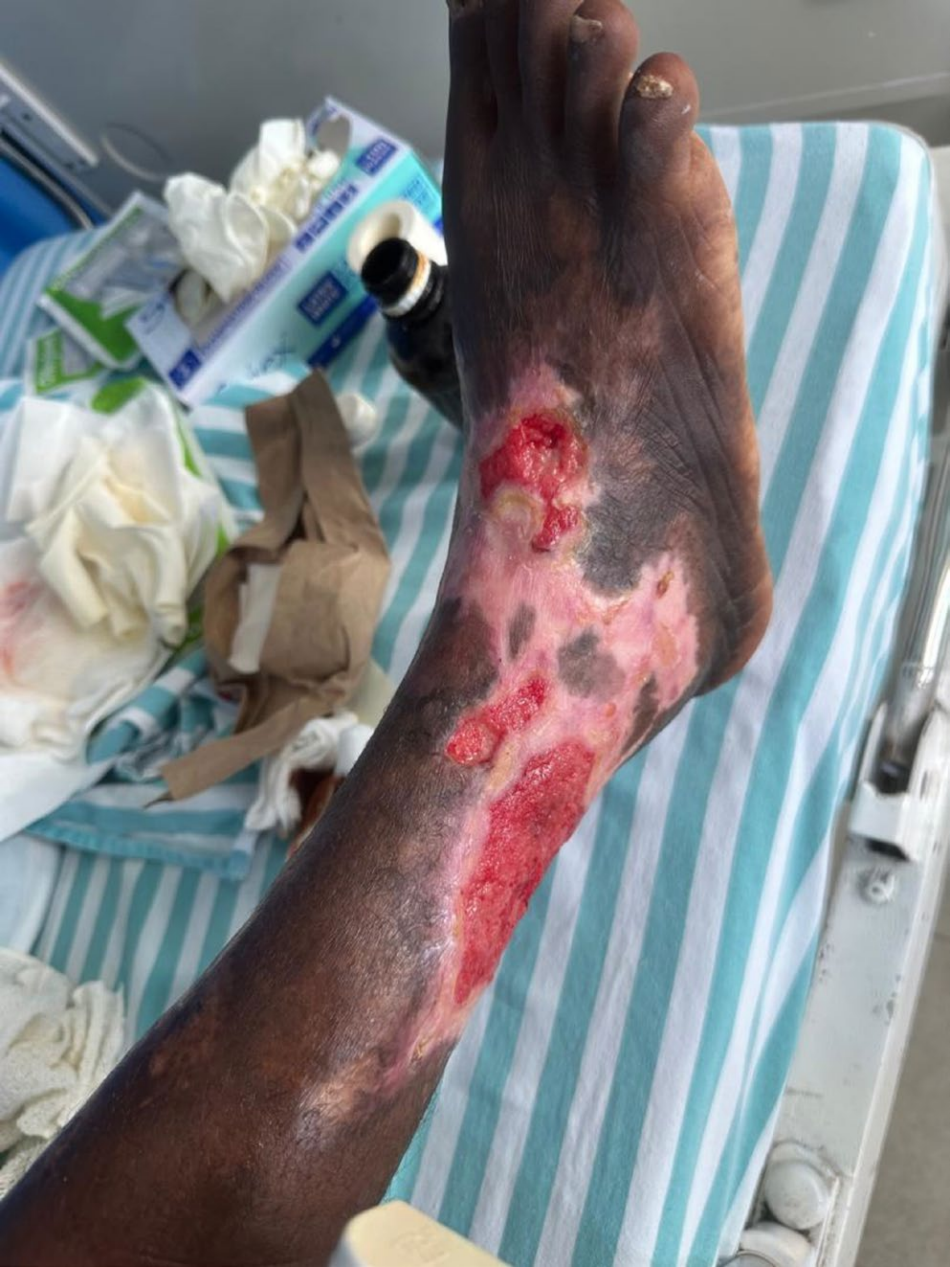
Gross photography depicting the largest leg ulcer measuring about 9 × 10 cm, located on the anterolateral aspect of the right leg extending to the dorsum of the foot with sloping edge and granulation tissue.

The lower extremities showed multiple ulcers with sloping edges and granulation tissue. The largest, measuring approximately 9 × 10 cm, was on the anterolateral aspect of the right leg, extending to the dorsum of the foot, with scant serous discharge and normal surrounding skin (Figure 2).

The patient had received empirical treatment at local health facilities, including antibiotics and wound dressings, with no significant improvement. Differential diagnoses considered included sexually transmitted infections, vasculitis, malignancy, and traumatic ulceration, all of which were ruled out by clinical evaluation and investigations.

Further assessment revealed no underlying immunosuppressive condition or systemic disease. The detailed progression of symptoms and prior treatment failures, along with clinical and histopathological findings, contributed to the diagnosis of penile pyoderma gangrenosum.

## 3 Methods (Investigations and Treatment)

Investigations revealed elevated CRP (133 mg/dL) and neutrophilia (7100/mm^3^). Tests for HIV, syphilis, and herpes simplex virus were conducted using ELISA for HIV, VDRL for syphilis, and PCR for HSV—all returned negative results. Fasting blood glucose was normal (4.3 mmol/L). Pus culture from the ulcers revealed gram‐positive bacteria with polymorphonuclear leukocytosis.

**FIGURE 3:**
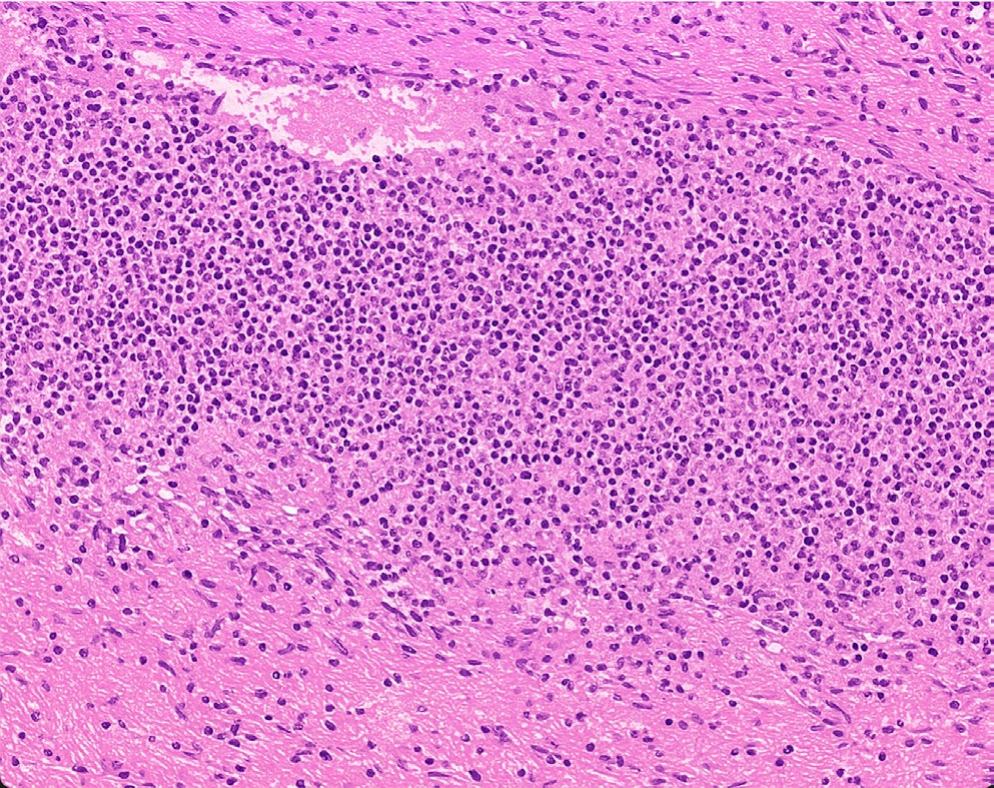
Histopathological examination of the penile lesion revealed nonspecific chronic inflammation with dense neutrophilic infiltration and tissue necrosis.

Histopathological examination of the penile lesion revealed nonspecific chronic inflammation with dense neutrophilic infiltration and tissue necrosis. A biopsy from the leg ulcers showed granulation tissue without evidence of malignancy or specific infection. (Figure 3 illustrates the histopathology slide).

**FIGURE 4:**
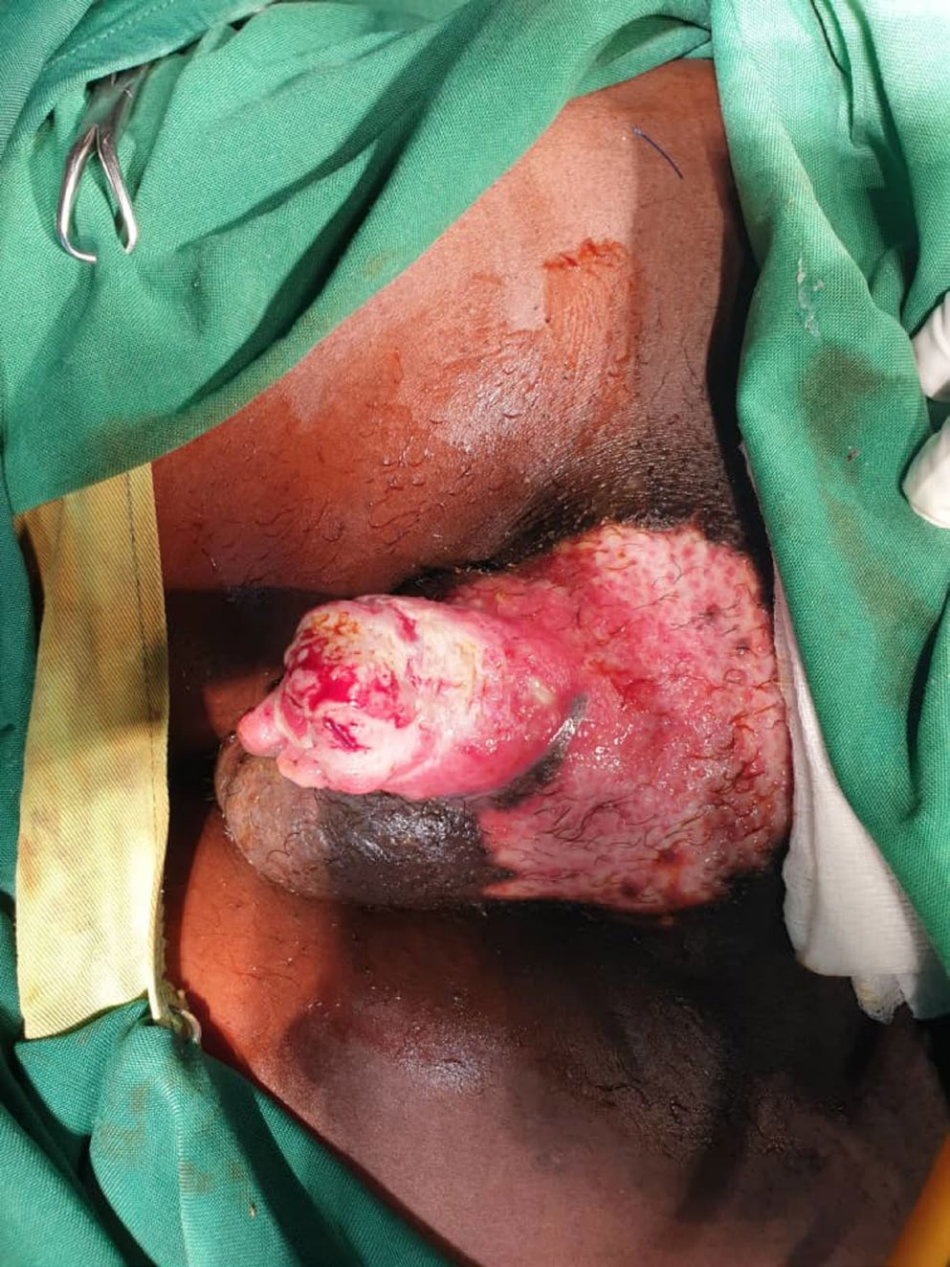
Gross photography depicting the genital ulcer following minimal sloughectomy and suprapubic cystostomy.

Based on clinical findings and biopsy results, a diagnosis of pyoderma gangrenosum was established. Initial management included suprapubic cystostomy for urinary diversion (Figure 4). Minimal sloughectomy was performed to remove necrotic tissue that was contributing to obstruction and infection risk. Although surgery is typically avoided in PG due to the risk of pathergy, it was deemed necessary and performed with minimal manipulation after dermatological consultation.

The patient was started on oral prednisolone at a dose of 40 mg daily. The dose was tapered by 10 mg per week over a 4‐week period. Supportive management included daily wound care with honey dressings. Honey was chosen due to its antimicrobial properties, its role in promoting moist wound healing, and its non‐adherent nature, which is beneficial in delicate ulcerated areas like the genital region.

## 4 Conclusion and Results

**FIGURE 5:**
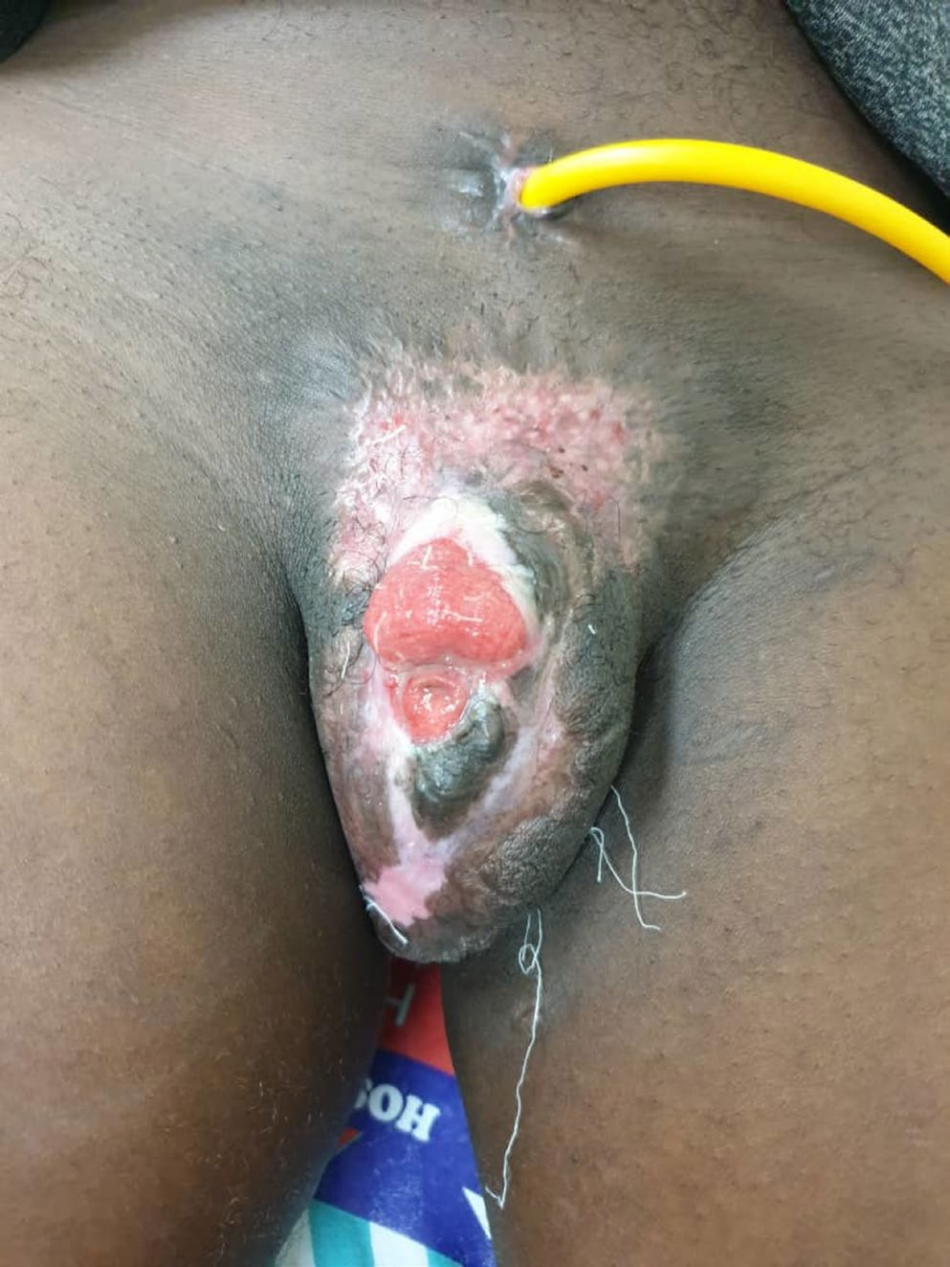
Gross photograph depicting the genital ulcer on day 21 following sloughectomy and initiation of oral prednisolone; nearly the whole ulcer has epithelized, sparing the tip of the penis.

The patient improved significantly within 3 days, with pain relief and epithelialization. By day eight, 50% of the ulcer had healed. At 3‐week follow‐up, the ulcer had nearly fully epithelialized (Figure 5).

## 5 Discussion

Pyoderma gangrenosum (PG) is a rare neutrophilic dermatosis that characteristically presents as painful, rapidly enlarging ulcers with irregular, undermined borders. It frequently coexists with systemic diseases such as inflammatory bowel disease (IBD), rheumatoid arthritis, and hematologic malignancies, although cases may occur in the absence of comorbid conditions [1, 2, 3]. Penile pyoderma gangrenosum (PPG) is an exceptionally rare manifestation, with fewer than 30 documented cases globally [4].

The pathogenesis of PG remains incompletely elucidated. It is thought to result from immune dysregulation involving aberrant neutrophil activation and recruitment, leading to uncontrolled inflammation and tissue destruction. Exacerbating triggers such as infections or trauma can initiate or worsen the lesions through a phenomenon known as pathergy [5, 6]. In PPG, the genital location contributes not only to physical debilitation but also to a significant psychological burden due to potential sexual dysfunction and the sensitive nature of the affected area [7, 8].

Diagnosis is primarily clinical and relies on the exclusion of infectious, neoplastic, and traumatic causes of genital ulceration. Histopathological features—predominantly dense neutrophilic infiltration with necrosis—provide supportive evidence but are not pathognomonic [9, 10]. Our patient underwent comprehensive evaluation, including biopsy and laboratory testing, which supported the diagnosis of PG. Although there is no definitive test, a consistent clinical picture, appropriate histology, and lack of response to antibiotics aid in distinguishing PG from mimics.

Systemic corticosteroids constitute the first‐line treatment and generally elicit rapid improvement. In steroid‐resistant cases, escalation to immunosuppressive therapies such as azathioprine, cyclosporine, or biologics may be warranted [11, 12]. Surgical intervention is typically discouraged due to the risk of pathergy; however, in our case, minimal sloughectomy was necessary to manage necrotic tissue contributing to obstruction and infection. This was performed cautiously and under dermatological guidance [13].

Honey dressings were employed as a nontraumatic, antimicrobial, and healing‐promoting modality. Their efficacy in chronic wounds and low propensity to aggravate lesions makes them a suitable adjunct in PG wound care [14].

Our case aligns with existing literature emphasizing early diagnosis and tailored immunosuppressive treatment in PPG. A review of comparable cases (Table 1) illustrates that conservative management often leads to favorable outcomes when instituted promptly. Timely therapeutic intervention, as demonstrated in our patient, mitigates complications such as scarring, mutilation, and erectile dysfunction [13, 15]. This case further highlights the importance of a multidisciplinary approach in managing complex dermatologic presentations involving urogenital structures.

**TABLE 1:** Summary of reported cases of penile pyoderma gangrenosum.

Author	Year	Age	Systemic disease	Treatment	Outcome
Khachemoune et al.	2012	38	None	Steroids	Resolved
Goldman et al.	2017	52	IBD	Steroids + cyclosporine	Resolved
Present case	2024	45	None	Steroids + cystostomy	Resolved

## 6 Conclusion

PPG should be considered in chronic genital ulcers unresponsive to antibiotics. Early steroid treatment and avoidance of extensive surgery can preserve function and improve outcomes.

## Author Contributions

**Shukrani John Ngereja:** conceptualization, writing – original draft. **Charles John Nhungo:** conceptualization, writing – original draft. **Ezekiel Petro:** writing – review and editing. **Thobias Mvungi:** writing – review and editing. **Ibrahim Ndolla:** writing – review and editing. **Muhaji Mohamed:** writing – review and editing. **Erasto Wambura:** writing – review and editing. **Mukama Kasori:** writing – review and editing. **Fransia Arda Mushi:** writing – review and editing. **Hamis Isaka:** writing – review and editing. **Obadia Nyongole:** writing – review and editing. **Charles Mkony:** writing – review and editing.

## Disclosure

This report has been published as per the CARE criteria.

## Ethics Statement

This case report study was exempt from ethical approval at our institution, as this paper reports a single case that emerged during normal surgical practice.

## Consent

Written informed consent was obtained from the patient for publication of this case report and accompanying images. A copy of the written patient consent is available for review by the Editor‐in‐Chief of this journal upon request.

## Conflicts of Interest

The authors declare no conflicts of interest.

## Data Availability

Due to privacy/ethical restrictions, the data supporting this case report are publicly not available.
